# Transcription factor network dynamics during the commitment to oncogene-induced senescence

**DOI:** 10.3389/freae.2024.1423454

**Published:** 2024-07-15

**Authors:** Themistoklis Vasilopoulos, Ricardo Iván Martínez-Zamudio

**Affiliations:** Robert Wood Johnson Medical School, Department of Pharmacology, Rutgers University, Piscataway, NJ, United States

**Keywords:** transcription factor networks, cell fate transition, OIS, multiomics, epigenome, highthroughput sequencing

## Abstract

Aberrant oncogenic signaling causes cells to transition into oncogene-induced senescence (OIS) to limit uncontrolled proliferation. Despite being a potent tumor suppressor mechanism, OIS is an unstable cell state susceptible to reprogramming that can promote tumorigenesis. Therefore, elucidating the underlying gene regulatory mechanisms that commit cells to OIS is critical to identifying actionable targets to modulate the senescence state. We previously showed that timely execution of the OIS program is governed by hierarchical transcription factor (TF) networks. However, the gene regulatory mechanisms that prime cells to commit to the OIS fate early upon oncogene hyperactivation are currently not known. Here, we leveraged our time-resolved multi-omic profiling approach to generate TF networks during the first 24 h of oncogenic HRAS^G12V^ activation. Using this approach, we demonstrate that the commitment to OIS requires the rearrangement of the TF network on a pre-established epigenomic landscape, priming the cells for the substantial chromatin remodeling that underpins the transition to OIS. Our results provide a detailed map of the chromatin landscape before cells transition to OIS thus offering a platform for manipulation of senescence outcomes of potentially therapeutic value.

## Introduction

Loss of cell identity (i.e., the differentiated phenotype) by oncogene hyperactivation, one of the earliest events during oncogenic transformation, occurs due to the destabilization of the transcription factor networks (Ji et al., 2021) that control cell type-specific gene expression (Wilkinson et al., 2017; Ferreiros et al., 2019). Aberrant oncogenic signaling can force cells with perturbed identities into oncogene-induced senescence (OIS), a potent tumor suppressor mechanism defined by a stable proliferative arrest and a pleiotropic senescence-associated secretory phenotype (SASP) (Braig et al., 2005; Bartkova et al., 2006; Campisi, 2013; Martinez-Zamudio et al., 2017). Despite the loss of proliferative capacity, cells in OIS remain highly dynamic entities which can reprogram themselves as well as destabilize the identity of cells within their surrounding tissue environment through cell autonomous and cell non-autonomous mechanisms (Lee and Schmitt, 2019). This functional heterogeneity of cells in OIS can lead to multiple, and sometimes paradoxical, outcomes, including reinforcement and destabilization of the proliferative arrest (Acosta et al., 2008; Martinez-Zamudio et al., 2023), reprogramming into a stem cell-like state (Milanovic et al., 2018) and transformation of non-malignant cells (Krtolica et al., 2001). Given their considerable impact on the outcome of carcinogenesis (Haugstetter et al., 2010; Eggert et al., 2016; Yu et al., 2018; Martinez-Zamudio et al., 2020; Martinez-Zamudio et al., 2023), understanding the mechanisms that commit cells to the OIS state early upon oncogene hyperactivation can lead to new therapeutic interventions of premalignant lesions.

Timing and duration of the oncogenic stimulus is critical for the commitment to the OIS phenotype (Wilson et al., 2017; Khaliq and Fallahi-Sichani, 2019). For instance, SASP from OIS fibroblasts has been shown to dynamically change its composition based on a NOTCH1-controlled switch that divides the secretome in two distinct phases post-induction of oncogenic RAS (Hoare et al., 2016). Additionally, it has been shown that the proliferative output of cells upon induction of BRAF^V600E^ can create population wide heterogeneity on the senescence outcome within the first 72 h (Chen et al., 2023). The transition to OIS is a highly organized, epigenetically precoded and reversible program that is mediated by hierarchical TF networks (Martinez-Zamudio et al., 2020). Yet, although the MAPK/ERK signaling cascade is activated within hours after RAS/BRAF activation (Chen et al., 2023), the duration of the oncogenic signal input and the time point at which cells commit to transition to OIS are unknown. Revealing the gene regulatory mechanisms that prime cells to commit to OIS can provide a detailed platform for manipulation of senescent cells with potentially therapeutic implications.

In this study, we defined the gene regulatory networks formed within the first 24 h of oncogenic HRAS^G12V^ activation that lead up to OIS commitment. We measured and integrated bulk transcriptome, epigenome and TF network dynamics at the early stages (0–24 h) upon oncogenic activation in the WI38 human lung fibroblasts undergoing HRAS-mediated OIS. This approach revealed that the TF network leverages a pre-established epigenomic landscape to promote the transcriptional changes required to transition to OIS within 24 h of oncogenic signaling.

## Results

### Dynamic multi-omic profiling to define the commitment to OIS

We performed time series experiments on human lung fibroblasts (strain WI38) undergoing OIS using the well characterized 4-hydroxytamoxifen (4-OHT)-inducible ER:RAS^G12V^ system (Young et al., 2009). We determined global transcriptomic, epigenomic and chromatin accessibility profiles using bulk RNA-seq, Cleavage Under Targets and Tagmentation (CUT&Tag) (Kaya-Okur et al., 2019) against H3K4me1 (putative enhancers), H3K27ac (activation) and H3K27me3 (facultative heterochromatin), and ATAC-seq at six time points (0, 2, 4, 12, 24, and 144 h after ER:RAS induction; Figure 1A). We verified the OIS state by monitoring morphological changes through light microscopy at each time point (Supplementary Figure S1A), RT-qPCR profiling of a panel of OIS-associated genes at 144 h post-induction as an end-point control (Supplementary Figure S1B), which we further confirmed by plotting their normalized counts at each time point (Supplementary Figure S1C). We confirmed activation of the RAS-MAPK pathway by immunoblotting against phosphorylated ERK1/2, which showed activation as early as 2 h after induction with 4-OHT**.** Levels of pERK1/2 peaked at 24 h post-induction and were maintained until the last time point at 144 h post-induction (see below).

**FIGURE 1:**
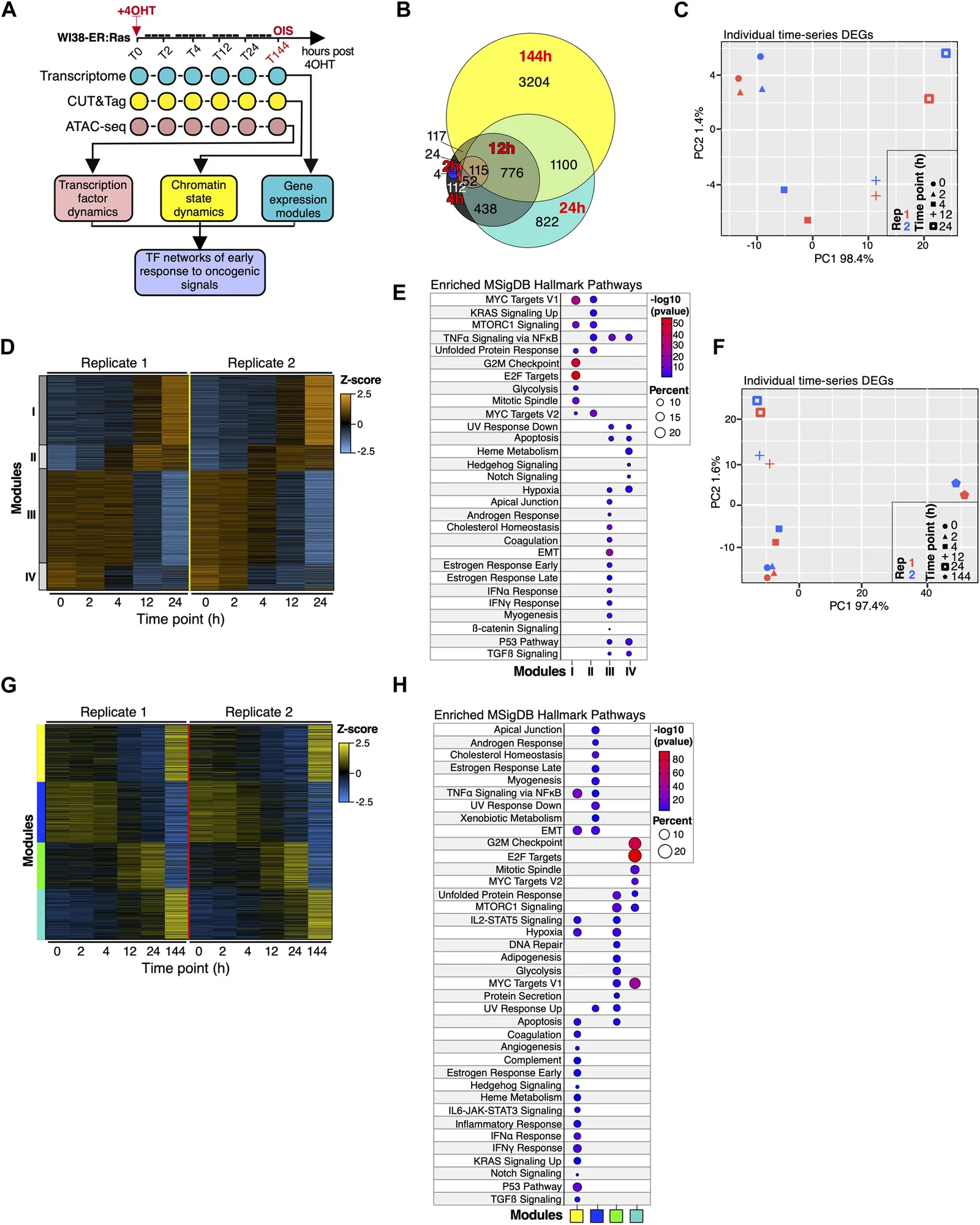
Transcriptional landscape of WI38 fibroblasts during the first 24 h of oncogenic HRAS^G12V^ overexpression. **(A)** Diagram describing the experimental approach for the time-resolved multi-omic profiling used to define the early gene-regulatory networks preceding OIS commitment. **(B)** Euler diagrams showing overlaps of differentially expressed genes (DEGs) for each time point in the time series relative to proliferating cells (0 h). **(C)** PCA projection plots showing the individual transcriptional trajectories of DEGs identified within 24 h of RAS induction. Two biologically independent time series are shown. **(D)** Heatmap showing the number-coded modules (I-IV) of DEGs identified within 24 h of RAS overexpression. Two biologically independent time series experiments are shown. **(E)** Functional over-representation analysis map showing significant associations of the Molecular Signatures Database (MSigDB) Hallmark gene sets for each module described in **(D)**. Circle fill is color-coded according to the FDR-corrected *p-value* from a hypergeometric distribution test. Circle size is proportional to the percentage of genes in each MSigDB gene set found within each gene module. *N* > 200 genes per module. **(F)** PCA projection plots showing the trajectories of DEGs identified through the entire time series (up to 144 h after RAS overexpression). **(G)** Heatmap showing the color-coded modules (yellow, blue, green and turquoise) of DEGs identified throughout the entire time series (up to 144 h after RAS overexpression). Two biologically independent time series experiments are shown. **(H)** Functional over-representation analysis map showing significant associations of the Molecular Signatures Database (MSigDB) Hallmark gene sets for each module described in **(G)**. Circle fill is color-coded according to the FDR-corrected *p-value* from a hypergeometric distribution test. Circle size is proportional to the percentage of genes in each MSigDB gene set found within each gene module. *N* > 200 genes per module.

### Early transcriptome dynamics precede commitment to OIS

To examine early gene expression dynamics occurring upon activation of the OIS program (up to 24 h post-induction), we applied principal component analysis and a machine learning self-organizing map (SOM) algorithm (Loffler-Wirth et al., 2015) to the transcriptomes of WI38 fibroblasts expressing HRAS^G12V^. These approaches revealed that cells rapidly activate a transcriptional response to HRAS^G12V^ expression, with detectable changes to the transcriptional trajectory as well as the SOM portraits as early as 4 h after HRAS^G12V^ induction, which continued to evolve until 24 h (Supplementary Figures S2A–D). Importantly, the transcriptional trajectories were remarkably reproducible across independent experiments. We identified ∼3,500 differentially expressed genes (DEGs) within 24 h upon HRAS^G12V^ activation (Figure 1B; Supplementary Table S1), whose transcriptional trajectory was virtually identical to that identified at the global transcriptome level (Figure 1C; Supplementary Figure S2A). We characterized the DEGs using weighted correlated gene network analysis (WGCNA) (Langfelder and Horvath, 2008), which identified 4 distinct modules (Figure 1D; Supplementary Table S1). Pathway analysis revealed upregulation of genes involved in RAS signaling, MYC targets, as well as TNF signaling (module II) all of which are associated with the activation of the MAPK/ERK pathway (Sabio and Davis, 2014), within 4 h of HRAS^G12V^ activation (Figure 1E). By 12 h, cells upregulate genes associated with mTORC1 signaling as well as G2M checkpoints (module I), indicating the cells are processing the aberrant oncogenic signal that perturbs the regulation of the cell cycle (Figure 1E) (Hsieh et al., 2018). Interestingly, genes in modules III and IV representing pathways playing major roles in senescence outcomes, such as the p53 pathway as well as SASP-associated pathways (interferon responses (IFNα/γ), transforming growth factor β (TGFβ) signaling and Notch signaling), are expressed in proliferating fibroblasts but become repressed by 24 h, indicating that a basal activity of these pathways is required to maintain cell identity which, upon HRAS^G12V^ induction, become repressed as the cells transition into OIS (see modules III, IV; Figure 1D). We then evaluated whether the transcriptional dynamics observed within 24 h of HRAS^G12V^ induction manifest in cells in OIS by reanalyzing the time series including the reference OIS time point of 144 h. To our surprise, inclusion of the 144 h time point resulted in the detection of an additional 3,204 DEGs (Figure 1B), which drove a drastic shift in the transcriptional trajectory of both DEGs and global transcriptomes between 24 h and 144 h (Figure 1F; Supplementary Figure S2E), consistent with our previous results (Martinez-Zamudio et al., 2020). WGCNA clustering identified 4 distinct modules (Figure 1G). Genes in the blue module, which enriched for pathways such as EMT, myogenesis and cholesterol homeostasis, were highly expressed in proliferating fibroblasts and early after HRAS^G12V^ induction, steadily decreasing until cells reached OIS (Figures 1G,H; Supplementary Table S1). Genes in the turquoise module, which steadily increased as the cells committed to OIS, were representative of cell cycle control and the metabolic sensor mTORC1 pathways, likely reflecting implementation of the cell cycle arrest and increased metabolic rates that are typical of senescent cells (Wiley and Campisi, 2021). Interestingly, genes in the yellow and green modules exhibited linear expression kinetics, decreasing and increasing, respectively, until 24 h after HRAS^G12V^ induction, then suddenly switching direction of expression, achieving maximal expression and repression, respectively, by 144 h (see yellow and green modules Figure 1G). Genes in the yellow module enriched for pathways characteristic of senescent cells, including inflammatory response, p53 pathway and various cytokine signaling pathways, while genes in the green module represented pathways characteristic of response to cell damage including DNA repair, apoptosis and UV response (Figure 1H). We confirmed the effect of the transcriptome at 144 h on the overall transcriptional trajectory as well as the switch in expression for a subset of gene clusters by SOM analysis (Supplementary Figures S2F–H; clusters D-I). To provide additional validation of the gene expression behavior during the first 24 h of oncogenic activation on a different cell line, we performed RT-qPCR profiling of a panel of OIS-associated gene in GM21-ER:RAS skin fibroblasts undergoing OIS. The pattern of expression of this OIS gene panel was comparable across two independent time series (Supplementary Figures S2I,J) and was also remarkably similar to normalized read counts for the same gene set in WI38-ER:RAS fibroblasts (compare to Supplementary Figure S1C). Together, these results highlight the highly organized nature of the early transcriptional events leading up to the commitment to OIS.

### Early gene regulatory events leading to the commitment to OIS take place in a pre-established chromatin landscape

Previous studies have shown a critical role of enhancers in the timely execution of the OIS program (Tasdemir et al., 2016; Guan et al., 2020; Martinez-Zamudio et al., 2020; Martinez-Zamudio et al., 2023). We therefore asked whether enhancer dynamics underpin the early transcriptional changes during the commitment to OIS. To this end, we initially focused on chromatin accessibility dynamics at H3K4me1-defined putative enhancers (Natoli, 2010; Heinz et al., 2015). PCA analysis of differentially accessible regions (DARs) (Figure 2A) matched those of DEGs and global transcriptomes. Surprisingly, despite the strong correlation between chromatin accessibility and transcriptional trajectories, only a modest amount of DARs was identified within 24 h of HRAS^G12V^ induction (244 DARs), with most chromatin accessibility changes occurring between 24h and 144 h (21,301 DARs) (Figure 2B; Supplementary Table S2). Clustering with WGCNA identified four modules (1–4) (Figure 2C; Supplementary Table S2). Modules 2 and 3 exhibited linear accessibility dynamics, closing and opening, respectively. Reminiscent of transcriptional modules, DAR modules 1 and 4 exhibited dynamic behavior, with increasing and decreasing accessibility up until 24 h, respectively, followed by a reversal of their accessibility by 144 h (Figure 2C). Similar results were obtained when chromatin accessibility datasets were analyzed in their totality (i.e., irrespective of histone modification-defined regulatory regions), with most chromatin accessibility changes occurring between 24 h and 144 h (Supplementary Figures S3A–C). Importantly, DARs, irrespective of the presence of H3K4me1, were prominently bound by AP1 family TFs prior to the induction of OIS (Figures 2D–F; Supplementary Figures S3D–F), confirming the critical role of these TFs in the execution of the OIS program (Han et al., 2018; Martinez-Zamudio et al., 2020). The limited chromatin accessibility changes occurring within 24 h of HRAS^G12V^ induction despite significant transcriptional dynamics within this time frame prompted us to evaluate the activation state of enhancers. We quantified the signal intensities of H3K4me1, H3K27ac and TF binding (ATAC) at opening, closing and dynamic enhancers throughout all timepoints. H3K4me1 levels remained stable for the duration of the experiment across all enhancer classes, as expected (Figures 2G–I, top panels). Surprisingly, basal levels of H3K27ac were detectable at all enhancer classes prior to RAS induction (Figures 2G–I, middle panels). The activation state of these enhancers varied modestly through the time series, reaching levels concordant with the accessibility state of each enhancer class once cells had achieved the OIS state (i.e., opening enhancers gained H3K27ac; closing lost H3K27ac; dynamic enhancers remained constant) (Figures 2G–I). This behavior was also observed for ATAC signals, which were readily detectable prior to RAS induction, remaining stable up until 24 h and followed by measurable gains or losses of chromatin binding by 144 h (Figures 2G–I, bottom panels).

**FIGURE 2:**
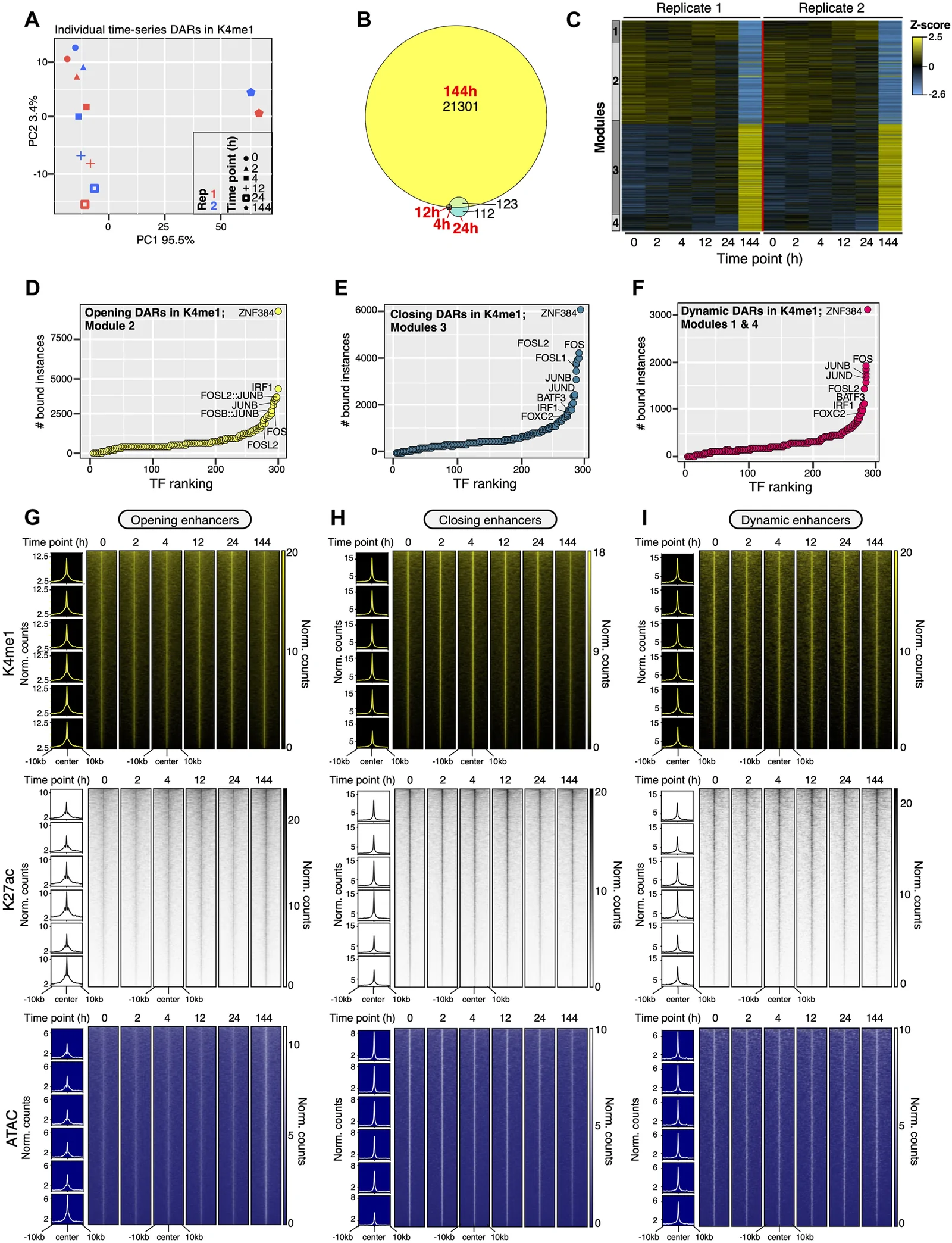
Gene regulatory events leading to commitment to OIS occur on a pre-established enhancer landscape. **(A)** PCA projection plots showing the individual trajectories of the differentially accessible regions (DARs) within putative enhancer regions (H3K4me1-defined enhancers). Two biologically independent time series are shown. **(B)** Euler diagram showing overlaps of DARs within putative enhancers regions for all time-points of the experiment up to 144 h. **(C)** Heatmap showing the number-coded modules (1–4) of DARs within putative enhancers per time-point as cells commit to OIS. The average of two biologically independent time series is shown. Modules represent DARs that are opening (Module 3), closing (Module 2) or demonstrate a more dynamic behavior (Modules 1, 4) throughout the time series. **(D–F)** Rank plots showing the summed binding instances of TFs at DARs within putative enhancers at each module defined in **(C)**. **(G–I)** Genome-wide signal intensity evolution heatmaps of H3K4me1, H3K27ac and ATAC-Seq signals in opening **(G)**, closing **(H)** and dynamic **(I)** putative enhancers as cells commit to OIS. The average of two independent time series is shown **(D–I)**. Insets to the left of heatmaps show the signal metaprofile of each respective histone modification and ATAC-seq signal in consecutive time points from top to bottom.

To gain further insight into the chromatin-based mechanisms leading to commitment to OIS, we performed a chromatin state transition analysis on our chromatin accessibility and histone modification datasets using the chromstaR algorithm. This approach maximizes detection of chromatin regions undergoing transitions through segmentation and quantification of non-overlapping featured-enriched regions at each time point (Hanna et al., 2018). Sixteen distinct chromatin states were identified, which were correctly annotated to their respective genomic regulatory regions in ENCODE project-defined chromatin states in normal human lung fibroblasts (NHLF) at each time point (i.e., H3K4me1 enriched at enhancers; H3K27me3 enriched at repressed regions; Supplementary Data). Consistent with our previous observations (Martinez-Zamudio et al., 2020; Martinez-Zamudio et al., 2023), the majority of the genome was devoid of histone modification or ATAC-seq signals (∼65%) (unmarked chromatin) followed by facultative heterochromatin (∼15%). Chromatin states linked to gene regulatory regions (enhancers and promoters) represented ∼20% collectively (Supplementary Figure S4A). Intriguingly, we did not find major chromatin state transitions within the first 24 h of HRAS^G12V^ induction. Most of the chromatin state transitions occurred between 24 h and 144 h, which largely involved activation of poised enhancers, commissioning of putative enhancers from unmarked chromatin and gains in TF binding at bivalent enhancers (defined as regions possessing H3K4me1, H3K27me3 and in some instances H3K27ac) (Blanco et al., 2020) (Figure 3A; Supplementary Figure S4B; Supplementary Table S3). Quantification of signal intensities of histone modifications and TF binding at each time point confirmed the results from the chromatin state transition analysis (Supplementary Figures S4C–E). The stability of the epigenome during the first 24 h upon induction of OIS seemed at odds with the measurable changes in gene expression during the same timeframe. To address this apparent incongruency, we integrated the top eight chromatin state transitions with the expression distribution dynamics of their nearby DEGs (Figure 3B). This analysis yielded several unexpected observations: I) the expression levels of DEGs near the top eight chromatin state transitions exhibited a relatively tight unimodal distribution during the first 12 h upon HRAS^G12V^ induction, II) as cells approach the transition point at 24 h, this unimodal distribution flattens, reflecting increased transcriptional noise (Tsuchiya et al., 2015) before III) committing to OIS by 144 h, at which time the distribution of DEG expression becomes bimodal, with gene expression reflecting its respective chromatin state transition (i.e., most genes near enhancers activated from a poised state become highly expressed in OIS cells) (Figure 3B; Supplementary Table S3). Correspondence analysis (CA) confirmed the coherence between chromatin state transitions and expression dynamics of DEG modules defined in Figure 1G (i.e., highly expressed genes in the turquoise module are closely associated with enhancer activation from the unmarked state upon commitment to OIS; Figure 3C). The early transcriptional dynamics in the absence of major chromatin remodeling suggests that early upon HRAS^G12V^ induction, gene expression is primarily controlled by TFs binding already accessible regions. This is consistent with the minor fraction of DARs observed within the first 24hrs in contrast to when the cells acquire OIS (Figure 2B; Supplementary Figure S3B). To identify potential TFs regulating chromatin state transitions during commitment to OIS, we performed footprinting analysis of unmarked, poised and bivalent enhancers prior to induction of HRAS^G12V^ (0 h) (Supplementary Table S3). We identified potential new players including IRF1, SOX15 and KLF family TFs as well as TFs involved in the regulation of OIS such as FOX family TFs and POU2F2 (Han et al., 2018; Martinez-Zamudio et al., 2023) (Figures 3D–F and see below). Overall, our comprehensive epigenomic analyses demonstrate that the early gene regulatory events leading to the commitment to OIS (within 24 h) occur within a pre-established chromatin landscape.

**FIGURE 3:**
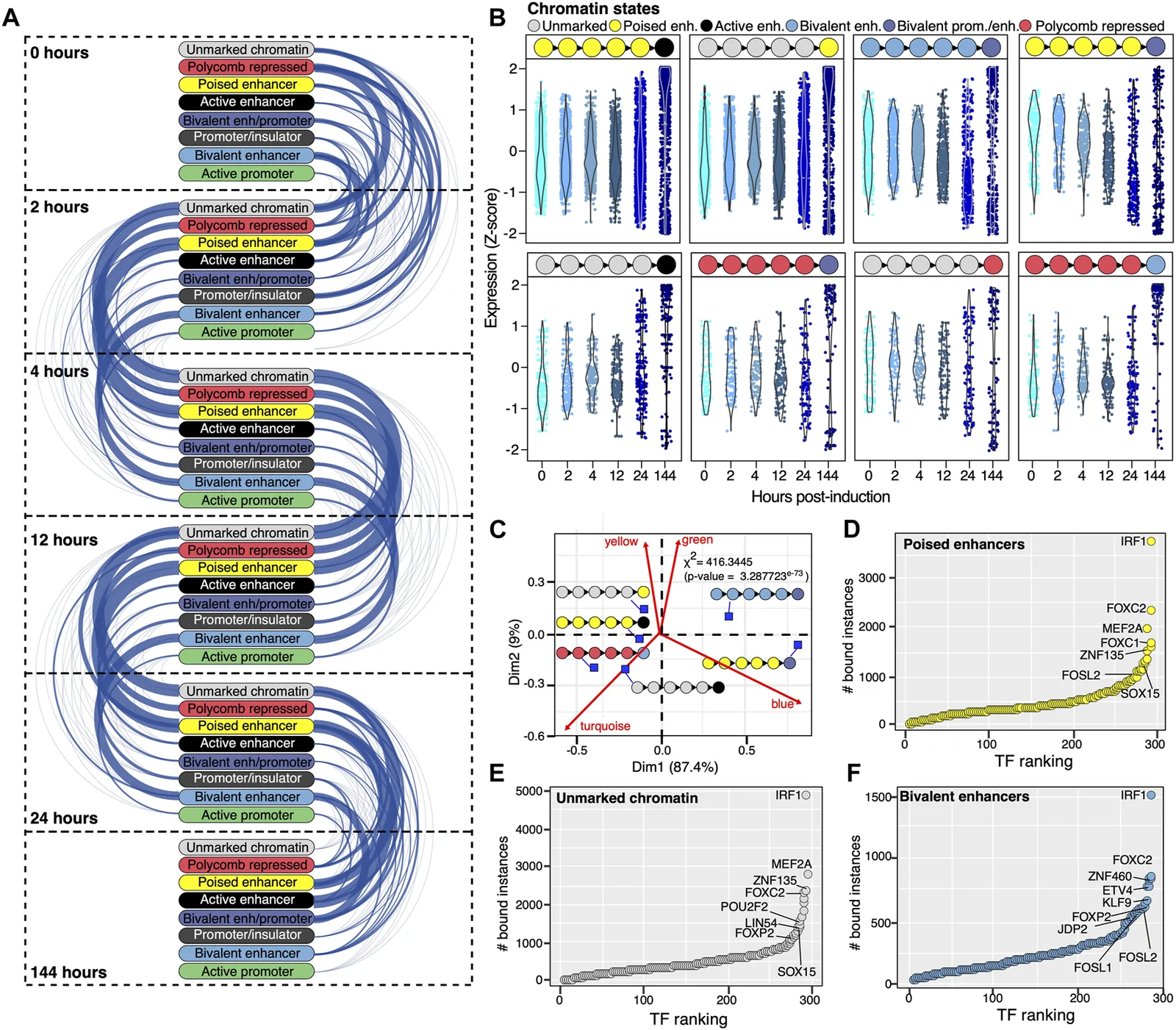
A stable epigenomic landscape precedes commitment to OIS. **(A)** Arc-plot visualization of eight select chromatin state transitions (each colored differently) at each indicated time-point during the first 24hrs post 4-OHT treatment and until OIS (144 h). The width of the edge is proportional to the number of 200 bp bins undergoing a given chromatin transition. **(B)** Integration of the top eight chromatin state transitions (top pictograms; 0 h–144 h from left to right) with nearby expression output of DEGs (Z score; violin plots; each dot represents a DEG). **(C)** Asymmetric biplot of the correspondence analysis (CA) showing the association between the top six, best-projected (squared cosine > 0.5), chromatin state transitions and DEG modules as defined in Figure 1G (blue, green, turquoise and yellow). The statistical significance of the association was calculated using a chi-squared test and shown as an inset. **(D–F)** Rank plots showing the summed binding instances of TFs at poised enhancers **(D)**, unmarked chromatin **(E)** and bivalent enhancers **(F)** prior to the induction of OIS (0 h). Chromatin state transitions, footprinting and expression data are the average of two independent time series.

### Widespread transcription factor network dynamics precede commitment to OIS

Given the limited changes to chromatin accessibility and chromatin states early upon HRAS^G12V^ induction, we explored the possibility that TF network activity may underlie the gene expression dynamics required for the commitment to OIS. To this end, we leveraged our TF footprinting datasets and initially determined TF co-binding interactions at DAR modules (Figure 4). We quantified TF binding instances identified at each point at closing, dynamic and opening DARs and subsequently projected them onto a co-binding matrix (Figures 4A–C). We identified AP1 family TFs as major mediators of TF interactions at all DAR classes, consistent with our previous findings (Martinez-Zamudio et al., 2023). In addition, we also detected additional co-binding clusters involving KLF, EGR and HOX family TFs. This was particularly notable at opening DARs, where widespread dynamic TF connectivity was observed (Figure 4C). Focusing the analysis on putative enhancers revealed similar results, albeit to a lesser degree (Supplementary Figures S5A–C). These data indicated that major TF network rearrangements are required during the early stages prior to the commitment to OIS. To address this possibility, we determined TF binding activity at all DAR classes (Li et al., 2019). Of note, widespread differential TF binding activity was readily detectable as early as 2 h upon induction of HRAS^G12V^ across all DAR classes and at putative enhancers (Figures 4D–F; Supplementary Figures S5D–F). Consistent with our transcriptome and epigenome analyses, the TF binding activity profile of cells in OIS was most dissimilar relative to those at earlier time points, particularly at opening DARs (and DARs at putative enhancers) where most of the TF network rearrangements were observed (Figures 4C, F; Supplementary Figures S5C, F). We observed similar TF binding activity dynamics at poised enhancers, unmarked chromatin and bivalent enhancers which underwent major transitions between 24 h and 144 h after HRAS^G12V^ induction (Supplementary Figure S6). Incorporating the transcriptional status of the TFs in the network revealed that, although the TF network dynamics can be attributed to differentially expressed TFs (see annotations on Figures 4D–F, Supplementary Figures S5D–F, S6), most of these TFs are already expressed prior to the induction of OIS (i.e., TFs in the yellow and blue modules), consistent with the notion that the commitment to OIS leverages a pre-established epigenomic landscape.

**FIGURE 4:**
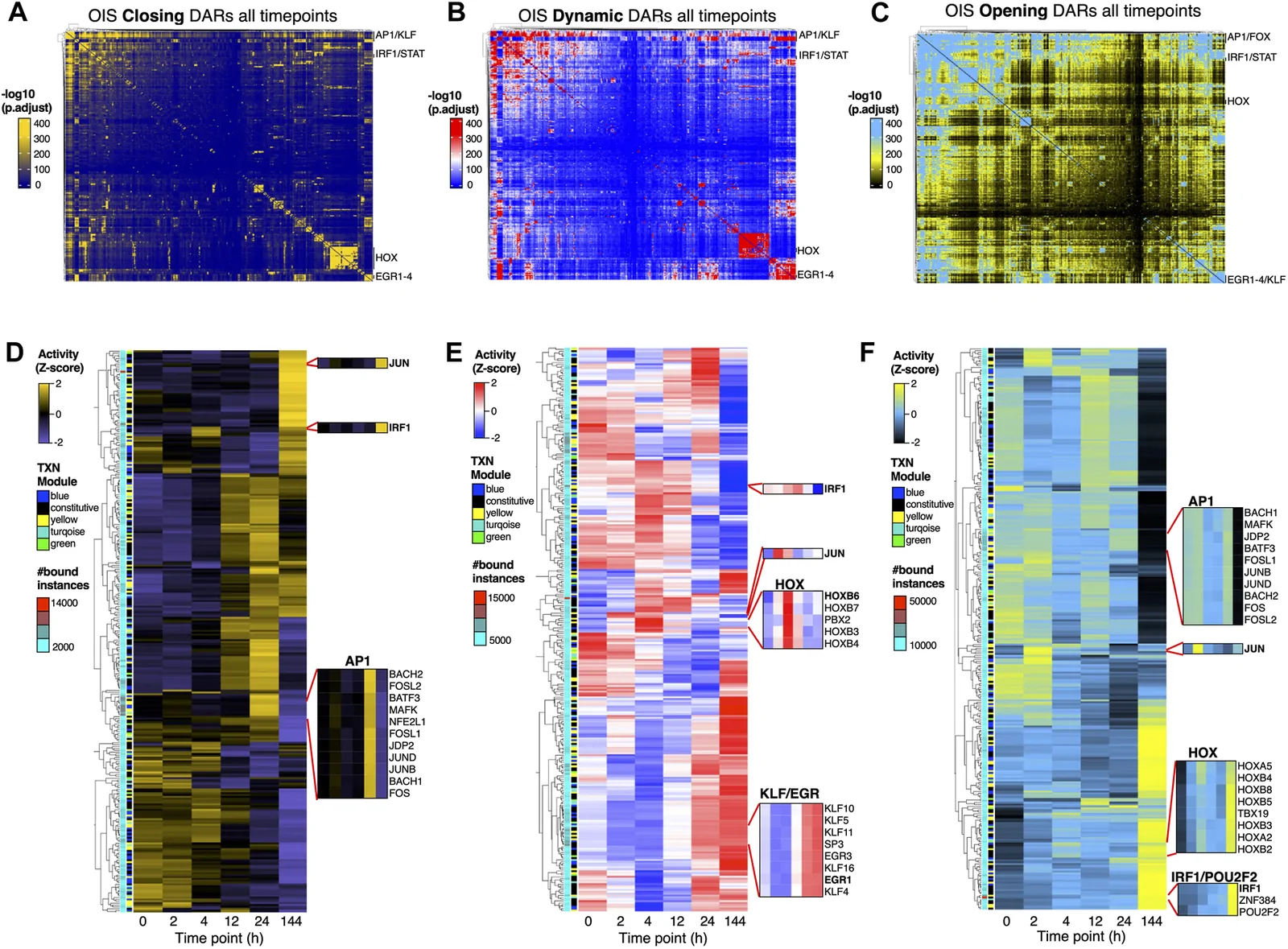
Widespread TF network rearrangements precede commitment to OIS. **(A–C)** Transcription factor co-binding matrices at closing **(A)**, dynamic **(B)** and opening **(C)** DARs (as defined in Supplementary Figure S3C modules) during the first 24 h and as cells enter OIS (144hrs). All binding instances across time points were collapsed onto the matrix and clustered using Ward’s aggregation criterion. The corresponding *q* values were projected onto the clustering and are color-coded based on significance calculated using a hypergeometric distribution test. **(D–F)** Heatmaps showing the differential TF chromatin binding activity (row Z-score) at closing **(D)**, dynamic **(E)** and opening **(F)** DARs for each time point. Only expressed TFs were considered in the analysis. The annotations on the left show the number of bound instances per TF and their gene expression (TXN) category (i.e., constitutively expressed [black] or differentially regulated according to the module color code shown in Figure 1G). Insets show the chromatin binding activity of representative TFs. TF footprinting **(A–F)** and differential chromatin binding activity were performed on ATAC-seq datasets from two biologically independent time series.

To visualize the dynamicity and structure of the TF interactome as cells transition to the OIS fate, we constructed TF networks focusing on the co-binding interactions at opening, dynamic and closing DARs (Figure 5; Supplementary Figure S7; Supplementary Table S4). In line with our previous work, the TF networks had a hierarchical structure composed of top, core and bottom layers (Figure 5; Supplementary Figure S7; Supplementary Table S4). However, and in contrast to our previous work at later time points during the commitment to and escape from OIS, the top layers of TF networks at opening and dynamic DARs were composed of numerous constitutively as well as differentially expressed TFs (202 TFs at opening DARs; 185 TFs at dynamic DARs) comprising multiple TF families including HOX, FOX, KLF, IRF, EGR as well as various ZNF TFs in addition to master regulators AP1 TFs (Figures 5A,B; Supplementary Table S4). In contrast, the top layer of the TF network at closing DARs featured exclusively AP1 TFs (Supplementary Figure S7; Supplementary Table S4). The increased complexity at the top layers at opening and dynamic DARs is reminiscent of the TF binding dynamics during embryonic stem (ES) cell differentiation, which, similar to findings presented here, precede the widespread chromatin remodeling upon germ layer commitment (Tsankov et al., 2015). To validate the observations from TF co-binding and activity studies, we monitored the protein levels of a subset of top layer, differentially expressed TFs; Interferon response factor 1 (IRF1), Early growth response 1 (EGR1) and Homeobox B6 (HOXB6) (Figure 5C; insets), which exhibited dynamic binding activity at DARs, putative enhancers, and at the top three most frequent chromatin state transitions (Figures 4D–F, Supplementary Figures S5, S6). As a reference, we also included the AP1 TF c-JUN. Immunoblot analysis showed strong basal expression for all TFs across independent experiments (Figure 5D). Upon induction of HRAS^G12V^, levels of EGR1, one of the early sensors of growth and stress signaling (Wang et al., 2021), were drastically reduced within 4 h–12 h, and remained at similar levels until the last time point at 144 h. Interestingly, EGR1 activity at DARs and putative enhancers was variable through the duration of the experiments (Figures 4D–F). Similarly, protein expression of IRF1, master regulator of the interferon response (Feng et al., 2021), exhibited cycles of down- and up-regulation, which generally matched its activity at genomic regulatory regions (Figures 4D–F; Figure 5D). Expression of HOXB6, a member of the developmental regulators HOX TFs (Smith et al., 2019; Steens and Klein, 2022), gradually increased until reaching maximal levels at 24 h post HRAS^G12V^ induction. Expectedly, the protein levels of c-JUN were essentially stable throughout the duration of the experiments (Figure 5D). The readily detectable basal expression of these and other TFs in our network highlight a widespread role for the TF network in the maintenance of the fibroblast cell identity (Wilkinson et al., 2017). Additionally, the ability of the cell to rapidly control both the expression and activity of nodes within the network in response to HRAS^G12V^ induction emphasizes its sensitivity to cell identity-perturbing stimuli. Collectively, our analyses of TF network dynamics confirm that a high degree of responsiveness of the pre-existing TF network to oncogenic stress induced by HRAS^G12V^ overexpression precedes the commitment to OIS.

**FIGURE 5:**
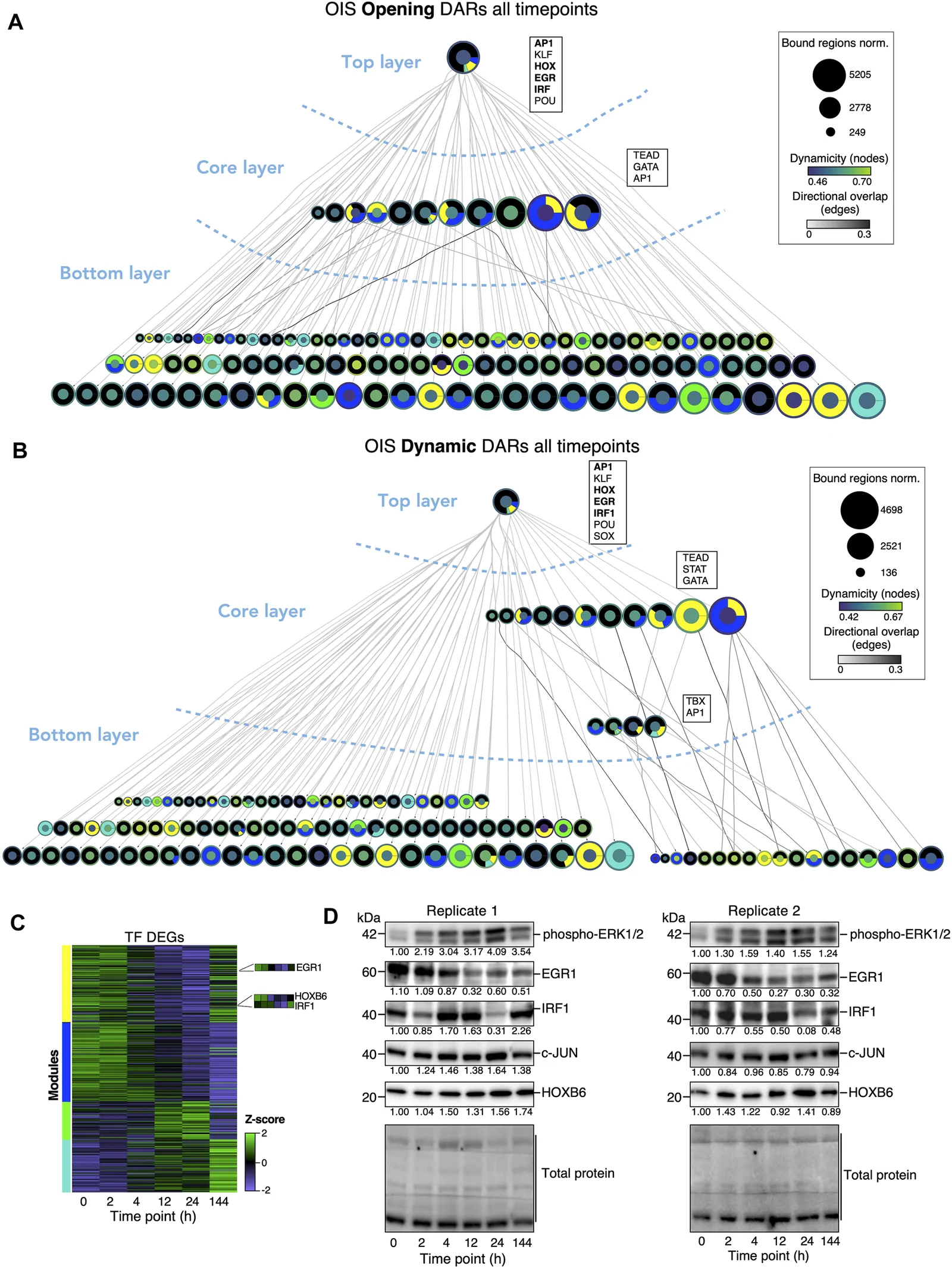
TF networks dynamics define the commitment to OIS. **(A, B)** TF networks at opening **(A)** and dynamic **(B)** DARs. TFs (nodes) are represented as circles. Oriented edges (arrows) connecting nodes indicate that at least 15% of the regions bound by a given TF in the bottom and core layers were bound by the interacting TF in the core and top layers, respectively, at the same or previous time points. Strongly connected components (SCCs) are represented as a single node to facilitate visualization. The fill color of the node’s inner circle is based on the normalized dynamicity of TFs. The fill color of the outer ring indicates whether the TF is constitutively expressed or belongs to a transcriptomic module (yellow, blue, green, turquoise). The node’s size is proportional to the bound regions by a given TF. Each network has three layers: i) the top layer with no incoming edges, ii) the core layer with incoming and outgoing edges, and iii) the bottom layer with no outgoing edges. Representative TF families at the top and core layers are shown (see text). Networks were generated from pooled ATAC-seq data sets from two biologically independent time series. **(C)** Heatmap showing the expression levels of genes encoding TFs at each DEG modules as defined in Figure 1G throughout the time series. **(D)** Western blot analysis of representative top layer, dynamic TFs (EGR1, IRF1, cJUN, HOXB6) as well as phorphorylated ERK at the indicated timepoints after induction of OIS. Relative quantification values of band density relative to T0 are shown below bands. Total protein staining of the blot was used as a loading control. Two biologically independent time series are shown. Data in **(C)** are averaged from two independent time series.

## Discussion

Although the tumor suppressive role of OIS has been well established, recent evidence shows that OIS can play a determinant role on the outcome of carcinogenesis via cell autonomous and cell non-autonomous mechanisms. For instance, the SASP generated by hepatocytes in OIS can promote the immune clearance of pre-malignant hepatocytes while simultaneously fueling growth of carcinoma cells through inhibition of the natural killer cell function (Eggert et al., 2016). Also, cells which have remained in OIS or therapy-induced senescence (TIS) for prolonged periods can undergo epigenetic reprogramming, resulting in TF network rearrangements and stemness reprogramming that facilitate senescence escape (Milanovic et al., 2018; Zampetidis et al., 2021; Martinez-Zamudio et al., 2023). Furthermore, the heterogeneity of OIS, which is dependent on the nature and duration of the oncogenic trigger as well as the tissue of origin, manifests at the single cell level and can impact cancer development at the subpopulation level (Chatsirisupachai et al., 2021). Thus, the continuous evolution of the OIS state determines the functional outcome in the progression of premalignant lesions. This heterogeneity of the OIS state has an outsized negative impact on the development of therapies that target senescent cells, which is reflected by an underwhelming efficacy of senotherapies (Dolgin, 2020). Under this light, understanding the gene regulatory mechanisms that commit cells to OIS may open new opportunities to develop more effective therapies to prevent cancer development by preventing the transition to this metastable cell state susceptible to reprogramming.

Here we defined the gene regulatory networks during the early time points prior to the commitment to OIS. By integrating gene expression, epigenome and TF binding dynamics we identified the time point at which cells under oncogenic stress due to HRAS^G12V^ overexpression prepare to commit to OIS. Based on our findings, we hypothesize this event occurs at 24 h post RAS induction, at which the transcriptional and chromatin accessibility trajectories of these cells undergo a drastic shift. From a gene expression point of view, this shift in trajectory coincides with the repression of DNA repair and metabolic genes and the upregulation of cell cycle control and SASP-associated genes between 24 h and 144 h after RAS induction. These events likely reflect an initial attempt of the cell to overcome the DNA damage incurred by HRAS^G12V^ overexpression, including replication stress (Fumagalli et al., 2014). As the cell’s machinery becomes insufficient to deal with the damage, the transition to OIS occurs, as reflected by the detection of an additional 3,204 DEGs exclusive to senescent cells.

In contrast, despite being virtually identical to transcriptional trajectories, only a small fraction of the accessible chromatin globally and at putative enhancers underwent detectable changes within 24 h, with the bulk of the chromatin accessibility changes occurring between 24 h and 144 h. Of note, putative enhancers were already bound by AP1 prior to the induction of oncogenic hyperactivity and basally activated, which subsequently underwent subtle but detectable (de)activation and gain/loss of TF binding as cells commit to OIS. Consistent with the stability of putative enhancers early upon induction of OIS, genomic regions undergoing chromatin state transitions were also remarkably stable within 24 h of HRAS^G12V^ induction. The most abundant transitions involved unmarked chromatin, poised and bivalent enhancers gaining substantial chromatin binding and acetylation between 24 h and 144 h, which coincided with the resolution of the directionality of expression of nearby genes. Thus, cells leverage and modulate their existing epigenomic landscape to prepare for the commitment to OIS. In the absence of a requirement of major chromatin remodeling, we identified extensive TF network rewiring occurring early upon HRAS^G12V^ overexpression. Indeed, in addition to the master regulators AP1, the cell modulates the expression and activity of various top layer, previously bound TFs, which subsequently drive the transcriptional and chromatin state changes required for commitment to OIS. Among these TFs, we validated the dynamic regulation of IRF1, EGR1 and HOXB6. Interestingly, IRF1 and EGR1 have been recently implicated with cellular senescence (Sadangi et al., 2022; Moiseeva et al., 2023). IRF1, a master regulator of the interferon pathway, has been reported to induce proliferative arrest and regulate SASP-mediated inflammatory responses (Moiseeva et al., 2023; Recchia Luciani et al., 2024). Given the dynamic regulation of IRF1 expression, with downregulation at 24 h and upregulation in senescent cells, we hypothesize that the interferon response may be required to commit cells to OIS, similar to cells undergoing replicative senescence (De Cecco et al., 2019). In contrast, EGR1, an early response TF, has been shown to act in a context-dependent manner playing roles both as a tumor suppressor and as an oncogene through direct regulation of p53 and TGFβ1 (Baron et al., 2006; Wang et al., 2021). Here, we show that EGR1 gets rapidly downregulated within 4 h post oncogenic induction and then stays stably expressed at low levels as cells become senescent (Figure 5C). Given its established role as a regulator of the MAPK pathway, the downregulation of EGR1 is surprising and suggests that its downregulation reflects an insult to cell identity. In line with this observation, it has been recently demonstrated that perturbance of EGR1 levels delayed the onset of B-RAF senescence in human skin fibroblasts (Carvalho et al., 2019)**.** This highly organized response of the TF network to a cell identity disrupting stimulus, previously described during the differentiation of ES cells (Tsankov et al., 2015), provides further evidence that OIS-associated cell fate transitions are the result of epigenetically precoded programs and not simple stress responses. Future work on the role of new TF network nodes in the commitment to OIS is required to identify actionable targets to manipulate this highly relevant cell state. In summary, this work provides further evidence of the dynamic yet highly organized nature of OIS and provides a logical framework for the modulation of senescence-associated cell fate transitions through manipulation of TF networks.

### Limitations of the study

While we provided a comprehensive description of the gene regulatory networks during the commitment to OIS, our data and particularly the lack of chromatin state transitions linked to transcriptional changes early upon induction of OIS, highlights the possibility of additional chromatin-based mechanisms that contribute to this process. For instance, we cannot exclude the possibility of modulation of the three-dimensional structure of chromatin, which has been recently shown to play a critical role in the differentiation of myeloid progenitors into neutrophils (Patta et al., 2024). Similarly, while we detected widespread rearrangements of the TF network early upon the induction of OIS, this activity did not result in any detectable changes to enhancer-linked histone modifications. One possibility that could potentially link TF network rearrangements to transcriptional changes during the early stages of OIS is the ability of TFs to recruit DNA demethylation machinery to prospective OIS enhancers, as has been previously described during the reprogramming of B cells into induced pluripotent stem cells (iPSCs) (Sardina et al., 2018). Incorporation of time resolved high-throughput chromosome conformation capture methods such as Hi-C, ChIA-PET and (oxidative)bisulphite-sequencing ([ox]BS-seq) will address these possibilities in future work.

## Methods

### Cell culture

WI38 normal human lung fibroblasts (CCL-75, ATCC -American Type Culture Collection) and GM21808 normal human skin fibroblasts (Coriell Institute) were cultured in a DMEM (D6429, Millipore-Sigma) medium supplemented with 10% fetal bovine serum, (#25–514, GenClone) at 37°C in a 5% CO_2_ and 2% O_2_ atmosphere. WI38-ER:RAS^G12V^ & GM21-ER:RAS^G12V^ cells have been generated via retroviral transduction as described in (Benhamed et al., 2012) using retroviral plasmid pLNCX2 ER:RAS (Young et al., 2009) (Addgene: 67844) and Platinum-A (Plat-A, Cell Biolabs) as the retroviral packaging cell line cultured in the same medium as WI38 cells. Once generated, WI38-ER:RAS^G12V^ fibroblasts were cultured as above and stimulated with 400 nM 4-hydroxytamoxifen (4-OHT)(H7904, Millipore-Sigma); samples were collected and processed at the time points indicated in the main text post treatment with 4-OHT. The 0 h uninduced control sample was treated with the same volume of EtOH (4-OHT vehicle) for 2 h. Cell images for phenotypic validation of OIS induction were obtained using an EVOS M5000 digital inverted microscope (Thermo Fisher Scientific).

### Western blotting

Protein extracts from samples from each timepoint were prepared in 1x CHAPS Lysis buffer (S7705, Millipore-Sigma) containing protease and phosphatase inhibitor cocktail (#78440, Thermo Fisher Scientific). Protein concentration was measured using Qubit™ Protein and Protein Broad Range (BR) Assay Kit (Q33212, Thermo Fisher Scientific) on a Qubit™ 4 Fluorometer (Thermo Fisher Scientific) according to manufacturer’s instructions. A total of 15 μgs of whole-cell lysates were resolved on 4% –12% Bis-Tris Plus precast gels (NW4125, Thermo Fisher Scientific) in 1X MOPS SDS running buffer (Thermo Fisher Scientific), and proteins were transferred to PVDF membranes via the iBlot 3 Western blot transfer system (Invitrogen/Thermo Fisher Scientific) according to manufacturer’s instructions. Total protein staining in post transfer membranes was done using No-Stain™ Protein Labeling Reagent (A44449, Thermo Fisher Scientific) according to manufacturer’s instructions. Membranes were blocked in 5% non-fat dry milk in 1x TBST (150 mM NaCl, 10 mM Tris–HCl, pH 8.0, 0.05% Tween 20) at room temperature for 1 h, then incubated with primary antibodies for 2 h at room temperature or at 4°C overnight with gentle agitation. Membranes were washed three times in 1 × TBST for 10 min with shaking, then incubated with secondary antibodies for 1 h shaking, washed three times in 1 × TBST for 10 min with shaking. All blots were imaged using Amersham™ Imager 680 (GE Life Sciences). The following primary antibodies and dilutions were used: pERK (Cell Signaling Technologies; Cat no. #4370; 1:1000 dilution), EGR-1 (Santa Cruz Biotechnology; Cat no. sc-101033; 1:500 dilution), IRF-1 (Santa Cruz Biotechnology; Cat no. sc-74530; 1:500 dilution), c-Jun (Santa Cruz Biotechnology; Cat no. sc-1694; 1:1000 dilution), HoxB6 (Santa Cruz Biotechnology; Cat no. sc-166950; 1:1000 dilution). The following antibodies were used as secondary: HRP-conjugated goat anti-rabbit (PerkinElmer; Cat no. NEF812001EA; 1:6000 dilution) or anti-mouse (Cell Signaling Technologies; Cat no. #70765; 1:3000 dilution). Relative quantification of band intensity for each blot was performed with background subtraction and normalized to total protein for each lane (most prominent band of the total protein was used) using Fiji (ImageJ) software Version: 2.14.0/1.54f.

### Real-time quantitative polymerase chain reaction for molecular validation of OIS induction

WI38-ER:Ras^G12V^ were induced with 4-OHT as described above and collected 144 h post treatment. Total RNA was isolated from cells with RNeasy Mini kit (Qiagen, Germantown, MD) according to the manufacturer’s instructions. As per the manufacturer’s instructions, reverse transcription of 500–1000 ng of total RNA was carried out using the iScript cDNA synthesis kit (Bio-rad). Quantitative real-time PCR (qPCR) was performed using primers (listed below) with the iTaq Universal SYBR Green Supermix (Bio-rad) on a CFX Opus 96 Real-Time PCR detection system (Bio-rad). Samples were analyzed in technical triplicates, and GADPH levels were used for normalization. The following Qiagen QuantiTect Primers were used: GADPH (QT00079247), (QT00008799), IL1B (QT00021385), IL8 (QT00000322), CDKN2A (QT00089964), CDKN1A (QT00062090), CCNA2 (QT00014798).

### CUT&Tag

We performed CUT&Tag on 100,000–200,000 cells per target per timepoint using the CUTANA CUT&Tag kit (Epicypher: 14–1102) using the following antibodies: Epicypher: H3K4me1 (Epicypher: 13–0057), H3K27me3 (Epicypher: 13–005), H3K27ac (Active Motif: 39,133) and rabbit IgG as a negative control (Epicypher: 13–0042), according to the manufacturer’s instructions. Libraries were amplified for 16 cycles and quality assessed using a TapeStation 4200 instrument. Libraries were paired-end sequenced on a Novaseq-X instrument. 10–20 million reads per library were used for downstream analyses.

### ATAC-seq

The transposition reaction and library construction were performed as previously described in the original publication (Buenrostro et al., 2013) using 50,000–100,000 cells from each time point of the OIS escape time series (two biological replicates). DNA fragments were extracted using a Qiagen MinElute kit (Qiagen) and libraries were produced by PCR amplification (7 cycles) of tagmented DNA using an NEB Next High-Fidelity 2× PCR Master Mix (New England Biolabs). Library quality was assessed using an TapeStation 4200 instrument (Applied Biosystems). Paired-end sequencing was performed in an Illumina Novaseq-X instrument. Typically, 30–50 million reads per library were required for downstream analyses.

### RNA-seq

RNA from 50,000–100,000 cells per time point was purified using a Macherey-Nagel RNA XS Plus kit according to the manufacturer’s instructions (Macherey-Nagel, Duren, Germany). RNA integrity was evaluated in a TapeStation 4200 instrument system and only RNA with an integrity number >= 9 was used for library preparation. Libraries were constructed using the SMARTer Stranded V2 (TakaraBio) according to the manufacturer’s instructions (TakaraBio). Paired-end sequencing was performed on an Illumina Novaseq-X instrument. At least 40 million reads per sample (20 million per strand) were obtained and used for downstream analyses.

### Preprocessing of high-throughput sequencing data

Paired-end (RNA-seq, CUT&Tag and ATAC-seq) reads were processed as we previously described (Martinez-Zamudio et al., 2023) aligned to the GRCh38.d1.v1 version of the human genome using bowtie2 (Langmead and Salzberg, 2012) using the local mode. Low-quality reads and adapters were removed using fastq-mcf v.1.0.5 and cutadapt. Alignments were further processed using samtools v.1.1.1, and PCR and optical duplicates were removed with PicardTools v.2.2.2. Enriched regions for histone modifications and ATAC-seq were identified using MACS v.3.0 (Liu, 2014) (macs3 callpeak—nomodel—shiftsize—shift-control—gsize hs -p 1e-3). The identified peaks were subsequently processed using the irreproducibility discovery rate (IDR) pipeline (Landt et al., 2012), generating time point-specific reproducible peak sets for histone modifications and ATAC-seq at each time point. A master peak set was constructed using a custom bedops script that merges common peaks between samples. For RNA-seq samples, reads were counted using summarized overlaps and normalized with DESeq2 for visualization.

### Chromatin state transitions

We analyzed the genome-wide combinations of H3K4me1, H3K27ac, H3K27me3 and ATAC-seq signals on IDR-controlled reproducible peaks of each time point using the chromstaR package. The algorithm was run on differential mode and configured to partition the genome in 200 bp non-overlapping bins and count the number of reads of histone modification/ATAC-seq mapping into each bin at the time points indicated in the main text and modeled using a univariate HMM based on a two-component mixture, the zero-inflated binomial distribution. Subsequently, a multivariate HMM assigns every bin in the genome to one of the multivariate components considering 2(6-time points x4 genomic enrichment variables [histone modifications and ATAC-seq]) possible states. We focused on robust transitions of an enrichment score of >= 1 for further analysis. Integration with gene expression data was achieved by annotating the nearest DEGs with ChIPSeeker (Yu et al., 2015) to the 8 most frequent chromatin state transitions and monitoring their expression using violin plots. The association between chromatin state transitions and DEG modules was performed through Correspondence analysis (CA) and visualized on an asymmetric biplot after filtering for the top contributing chromatin state transitions (square cosine >0.5).

### Heatmap and metaprofile visualizations of ATAC-seq and CUT&Tag datasets

ATAC-seq and CUT&Tag alignments were normalized using deeptools v3.3.1100103 (Ramirez et al., 2016) using the RPGC approach to obtain 1X coverage (bamCoverage -b–normalizeUsing RPGC–effectiveGenomeSize 2864785220 –ignoreDuplicates–binSize 10 –verbose -o).

### Differential expression and accessibility

Differentially accessible regions (DARs) and differentially expressed genes (DEGs) throughout the time course were identified from ATAC-seq and RNA-seq data, respectively using DESeq2. Raw reads are internally normalized by DESeq2 using the median of ratios method (Anders and Huber, 2010). Reads per peak (ATAC-seq, using a custom master peak list as feature input) or exon (RNA-seq, using the GRCh38.107 genome model) were quantified using the *summarizeOverlaps* package and peaks/genes with at least 10 reads in at least 10 libraries were kept. Correction of batch effects was performed with limma (Ritchie et al., 2015) using “replicate” as the surrogate variable. Data transformation (regularized-log [rld] transformation), exploratory visualization (PCA and hierarchical clustering) and differential expression analysis were performed with DESeq2 using the default parameters as previously described (Love et al., 2014) and base R functions. We focused on highly significant DARs and DEGs by using an adjusted *p*-value filter of 0.05.

### WGCNA

Differentially expressed genes (DEGs) and accessible regions (DARs) identified with DESeq2 were used as input for unsupervised clustering using WGCNA. We used the “signed” option with default parameters, with the exception of the soft thresholding power which was set to 12 for RNA-seq data (0 h–24 h), 14 for RNA-seq data (all time points) and 12 for ATAC-seq data (accessible chromatin and H3K4me1-defined regions). The minimum size for the DEG and DAR modules was set to 200 features for the initial set of modules, which were then merged by a dissimilarity threshold of 0.3 for RNA-seq data and 0.08 for ATAC-seq data. For RNA-seq, WGCNA modules were functionally profiled using clusterProfiler (Wu et al., 2021) using the Molecular Signatures Database Hallmark gene sets (Liberzon et al., 2015). Statistical significance was calculated by a hypergeometric test with a cut-off of an adjusted *p*-value of <= 0.1 with Benjamini–Hochberg correction.

### Transcription factor footprinting

Footprinting, TF motif enrichment, and differential binding activity were performed as we previously described with HINT-ATAC (Li et al., 2019) using the JASPAR position weight matrix database for vertebrate TFs (Fornes et al., 2020) on merged ATAC-seq datasets, focusing on enhancer and global DARs as well as the 3 most frequent chromatin state transitions. To investigate TF co-binding, we joined chromatin regions containing TF binding instances less than 500 bp apart, resulting in a set of chromatin regions with variable sizes concentrating TF activity during the entire time course. We performed a hypergeometric test for each pairwise co-binding possibility for each time point and adjusted *p*-values for multiple testing using Bonferroni correction.

### Transcription factor networks

We built TF hierarchical networks as previously described in (Martinez-Zamudio et al., 2023), at opening, closing and dynamic DARs. Each identified TF is represented as a node, connected by directed edges representing co-binding events along the chromatin and during the entire time course. An edge with TF A as the source and TF B as the target indicates that TF A binds to the same chromatin regions as TF B at the same or at a previous time point. For each TF, we computed their normalized total number of bound regions and dynamicity and represented those properties in the networks as node size and node color, respectively. Each edge is associated with a weight in the interval [0, 1], representing the fraction of TF B binding instances previously occupied by TF A. We filtered the networks for edges with a weight higher than 0.15 and performed a transitive reduction to simplify the obtained networks while keeping essential topological features. Nodes included in the same strongly connected component (SCC), i.e., connected both by incoming and outgoing paths, were merged into a single node. We represented the identified transcriptomic module distribution for TFs included in the same SCC as Doughnut Charts.

## Data Availability

The data presented in the study are deposited in the GEO repository, accession numbers are GSE271459, GSE271460 and GSE271461.

## Supplementary material

SUPPLEMENTARY FIGURE S1 Validation of the WI38-ER:RASG12V cell system for induction of OIS. **(A)** Representative micrographs of proliferating (2 h post addition of vehicle [EtOH]) and senescent (144h post addition of 4-OHT) WI38 fibroblasts. Scale bar: 300 µm. **(B)** RT-qPCR profiling of senescence biomarkers for each individual biological replicate of time series (n = 2) comparing WI38-ER:RAS cells 144 h post treatment with EtOH (Proliferating) and 4-OHT (OIS). All targets were normalized using GAPDH as a reference gene. **(C)** Histogram showing the normalized reads for the indicated senescence biomarkers for each time point in WI38-ER:RAS^G12V^ fibroblasts undergoing OIS. The bars indicated the range of read counts per indicated gene (n = 2 biologically independent time series).

SUPPLEMENTARY FIGURE S2 Transcriptional landscape of WI38 fibroblasts during the first 24 h of oncogenic RAS overexpression. **(A)** PCA projection plots showing the individual global transcriptional trajectories of two biologically independent early time series during the first 24 h. **(B)** Averaged self-organizing maps (SOMs) of global transcriptomes of two biologically independent time series of WI38 fibroblasts during the first 24 h upon RAS activation, expressed as logarithmic fold-change. **(C)** D-cluster projection showing regions of SOMs with significant changes in expression during the first 24 h (see regions of SOMs in **(B)** that change color through time). **(D)** Expression of D-cluster metagenes in WI38 fibroblasts for every time point during the first 24hrs post RAS overexpression. The histograms show the change in the expression of metagenes in clusters identified in **(C)** (top of each panel). They are color-coded according to time point (bottom of panels). **(E)** PCA projection plots as in (A) through the entire time series (up to 144 h). **(F–H)** Averaged SOMs like, D-cluster projection and expression of respective metagenes as in (B-D) through the entire time series (up to 144 h). **(I,J)** RT-qPCR profiling of the indicated senescence biomarkers in GM21-ER:RAS^G12V^ skin fibroblasts undergoing OIS (n = 2 biologically independent time series).

SUPPLEMENTARY FIGURE S3 Gene regulatory events leading to commitment to OIS occur on a pre-established chromatin accessibility landscape. **(A)** Principal component analysis projection plots showing the individual trajectories of the differentially accessible regions (DARs) as assessed by ATAC-Seq of every time-point of two biologically independent time series. **(B)** Venn diagram showing intersections of DARs in **(A)** for all time-points of the experiment up to 144 h. **(C)** Heatmap showing the letter-coded modules **(A–C)** of DARs of WI38-ERRAS fibroblasts for every time-point through the entire time-course. The average of two biologically independent time series experiments is shown. Modules represent DARs that are opening throughout the duration of the time-course (Module A), closing (Module B) or demonstrate a more dynamic behavior (Modules C). **(D–F)** Rank plots showing the summed binding instances of TFs in chromatin regions of the modules defined in **(C)** of Opening **(D)**, Closing **(E)** and Dynamic **(F)** DARs.

SUPPLEMENTARY FIGURE S4 A stable epigenomic landscape precedes commitment to OIS. **(A)** Histograms showing the percentage of the genome enriched in the indicated combinations of H3K4me1, H3K27ac, H3K27me3 from CUT&Tag and ATAC-seq signals at the indicated time points after induction of OIS. **(B)** Quantification of the top 8 most frequent chromatin state transitions occurring through the time series. **(C–E)** Genome-wide signal intensity evolution heatmaps of H3K4me1, H3K27ac, H3K27me3 and ATAC-Seq signals at unmarked chromatin **(C)**, poised **(D)** and bivalent **(E)** enhancers identified prior to the induction of OIS (0 h) during the span of the time series. The average of two independent is shown. Insets to the left of heatmaps show the signal metaprofile of each respective histone modification and ATAC-seq signal in consecutive time points from top to bottom.

SUPPLEMENTARY FIGURE S5 Widespread TF network rearrangements precede commitment to OIS. **(A–C)** Transcription factor co-binding matrices at closing **(A)**, dynamic **(B)** and opening **(C)** putative enhancers (as defined in Figure 2C modules) during the first 24 h and as cells enter OIS (144 h). All binding instances across time points were collapsed onto the matrix and clustered using Ward’s aggregation criterion. The corresponding *q* values were projected onto the clustering and are color-coded based on significance calculated using a hypergeometric distribution test. **(D–F)** Heatmaps showing the differential TF chromatin binding activity (row Z-score) at closing **(D)**, dynamic **(E)** and opening **(F)** putative enhancers for each time point. Only expressed TFs were considered in the analysis. The annotations on the left show the number of bound instances per TF and their gene expression (TXN) category (i.e., constitutively expressed [black] or differentially regulated according to the module color code shown in Figure 1G). Insets show the chromatin binding activity of representative TFs. TF footprinting (A-F) and differential chromatin binding activity were performed on ATAC-seq/CUT&Tag (H3K4me1) datasets from two biologically independent time series.

SUPPLEMENTARY FIGURE S6 Widespread TF network rearrangements precede commitment to OIS. Heatmaps showing the differential TF chromatin binding activity (row Z-score) at poised enhancers **(A)**, unmarked chromatin **(B)** and bivalent enhancers **(C)** (as defined in Figures 3D–F) for each time point. Only expressed TFs were considered in the analysis. The annotations on the left show the number of bound instances per TF and their gene expression (TXN) category (i.e., constitutively expressed [black] or differentially regulated according to the module color code shown in Figure 1G). Insets show the chromatin binding activity of representative TFs. TF footprinting **(A–F)** and differential chromatin binding activity were performed on ATAC-seq/CUT&Tag datasets from two biologically independent time series.

SUPPLEMENTARY FIGURE S7 TF networks dynamics define the commitment to OIS. TF networks at closing DARs. TFs (nodes) are represented as circles. Oriented edges (arrows) connecting nodes indicate that at least 15% of the regions bound by a given TF in the bottom and core layers were bound by the interacting TF in the core and top layers, respectively, at the same or previous time points. Strongly connected components (SCCs) are represented as a single node to facilitate visualization. The fill color of the node’s inner circle is based on the normalized dynamicity of TFs. The fill color of the outer ring indicates whether the TF is constitutively expressed or belongs to a transcriptomic module (yellow, blue, green, turquoise). The node’s size is proportional to the bound regions by a given TF. Each network has three layers: i) the top layer with no incoming edges, ii) the core layer with incoming and outgoing edges, and iii) the bottom layer with no outgoing edges. Representative TF families at the top and core layers are shown (see text). Networks were generated from pooled ATAC-seq data sets from two biologically independent time series.

SUPPLEMENTARY DATA Enrichment heatmap for all time points of two averaged biologically independent time series showing the combinations of H3K4me1, H3K27ac, H3K27me3 CUT&Tag and ATAC-seq signals with reference chromatin states in normal human lung fibroblasts from the ENCODE project. Txn, transcription; CNV, copy number variation.
