# Supplementary material for: Transcription factor network dynamics during the commitment to oncogene-induced senescence
Source: Front Epigenet Epigenom. 2024 Jul 15;2:1423454. doi: 10.3389/freae.2024.1423454 (PMC13639277; doi:10.3389/freae.2024.1423454)
Supplement: Supplementary file 1 [file DataSheet1.PDF]

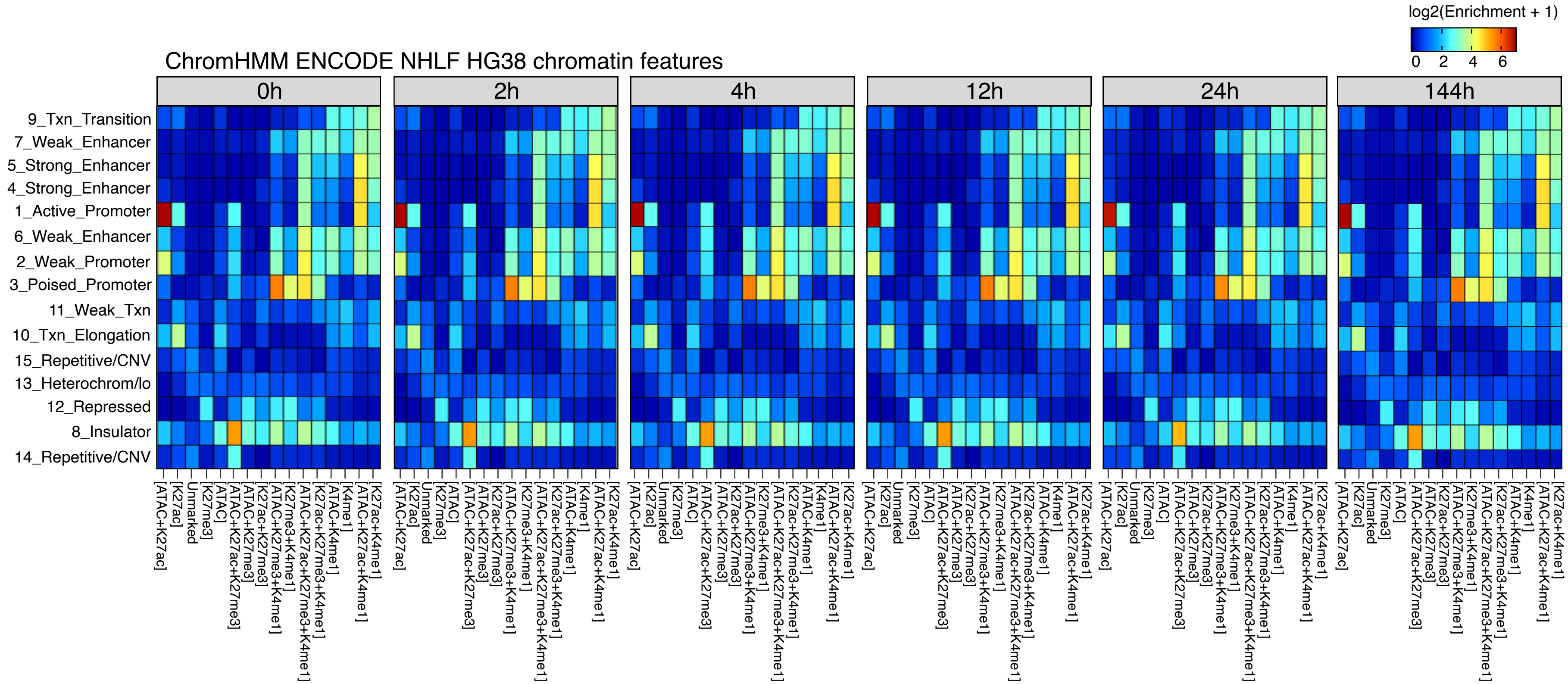

Supplementary Data
