# Supplementary material for: Transcription factor network dynamics during the commitment to oncogene-induced senescence
Source: Front Epigenet Epigenom. 2024 Jul 15;2:1423454. doi: 10.3389/freae.2024.1423454 (PMC13639277; doi:10.3389/freae.2024.1423454)
Supplement: Supplementary file 2 [file DataSheet2.pdf]

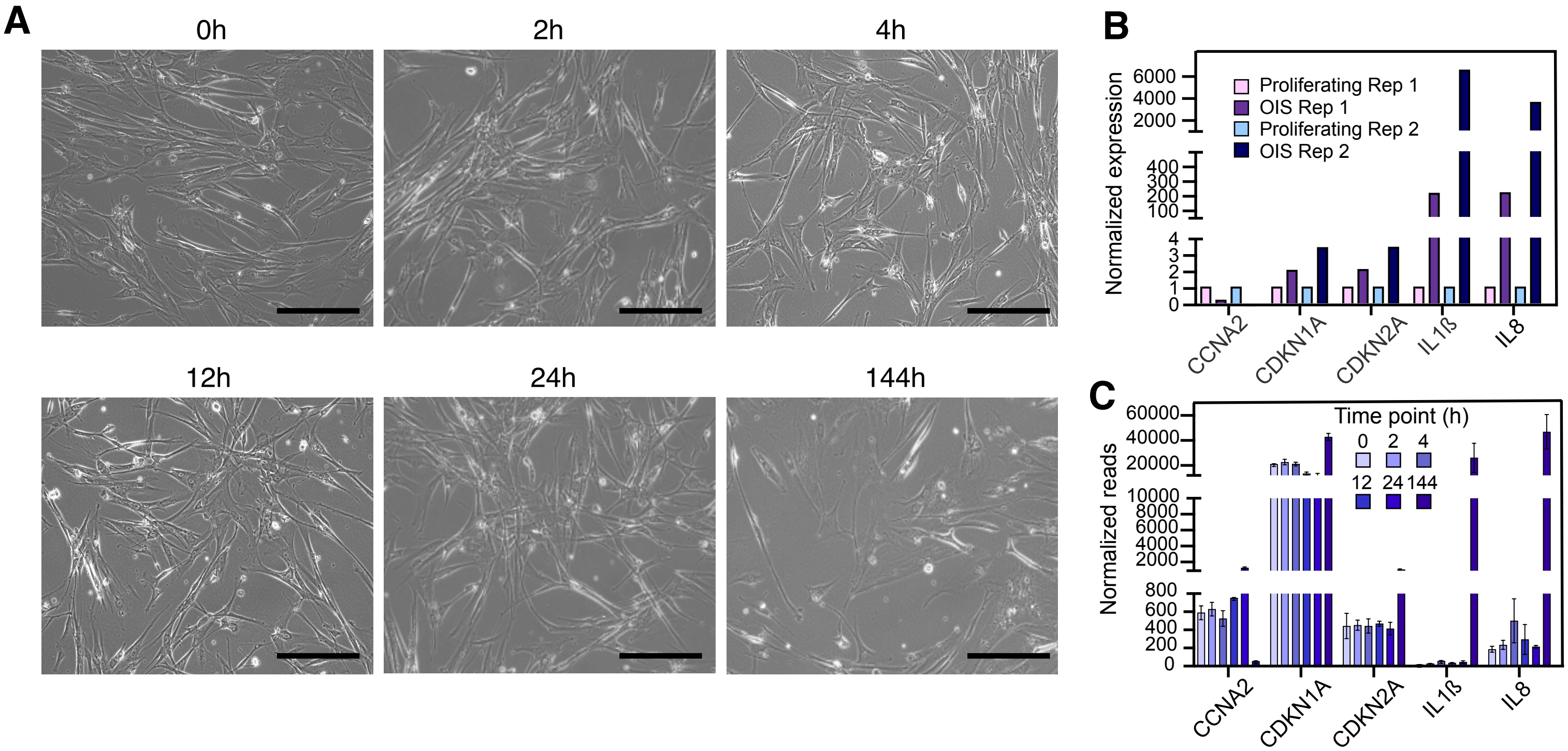

Supplementary Figure S1

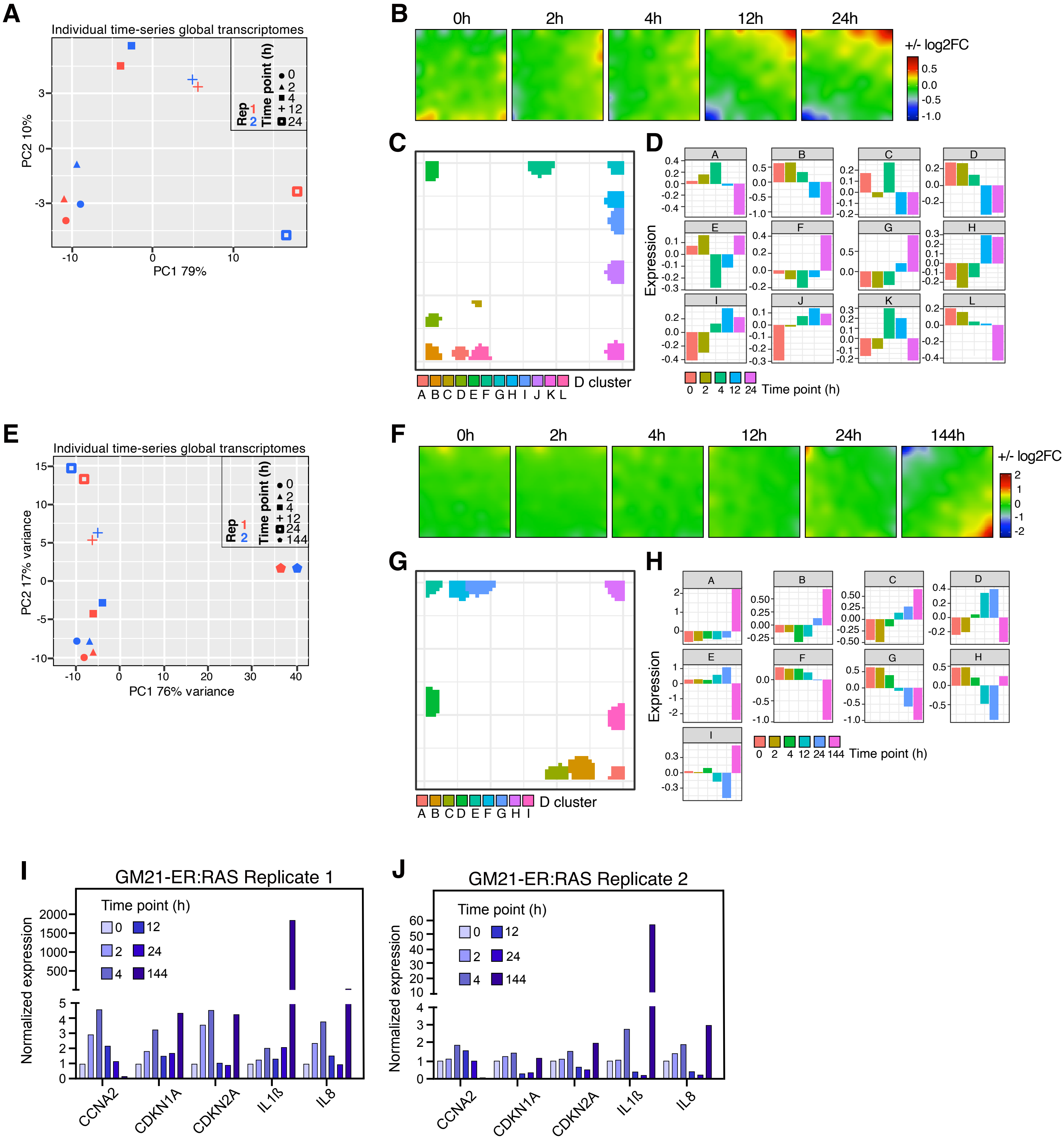

Supplementary Figure S2

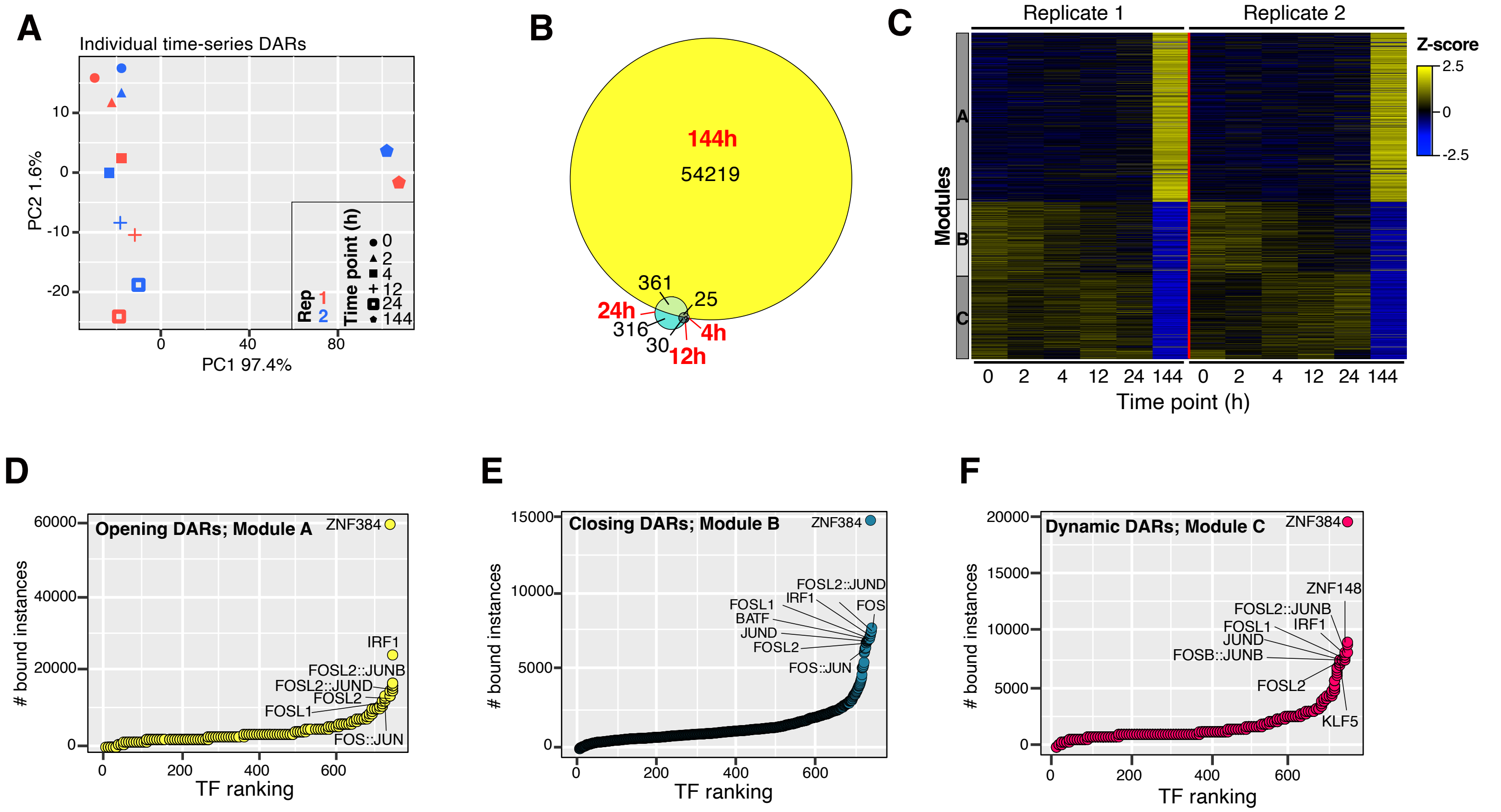

Supplementary Figure S3

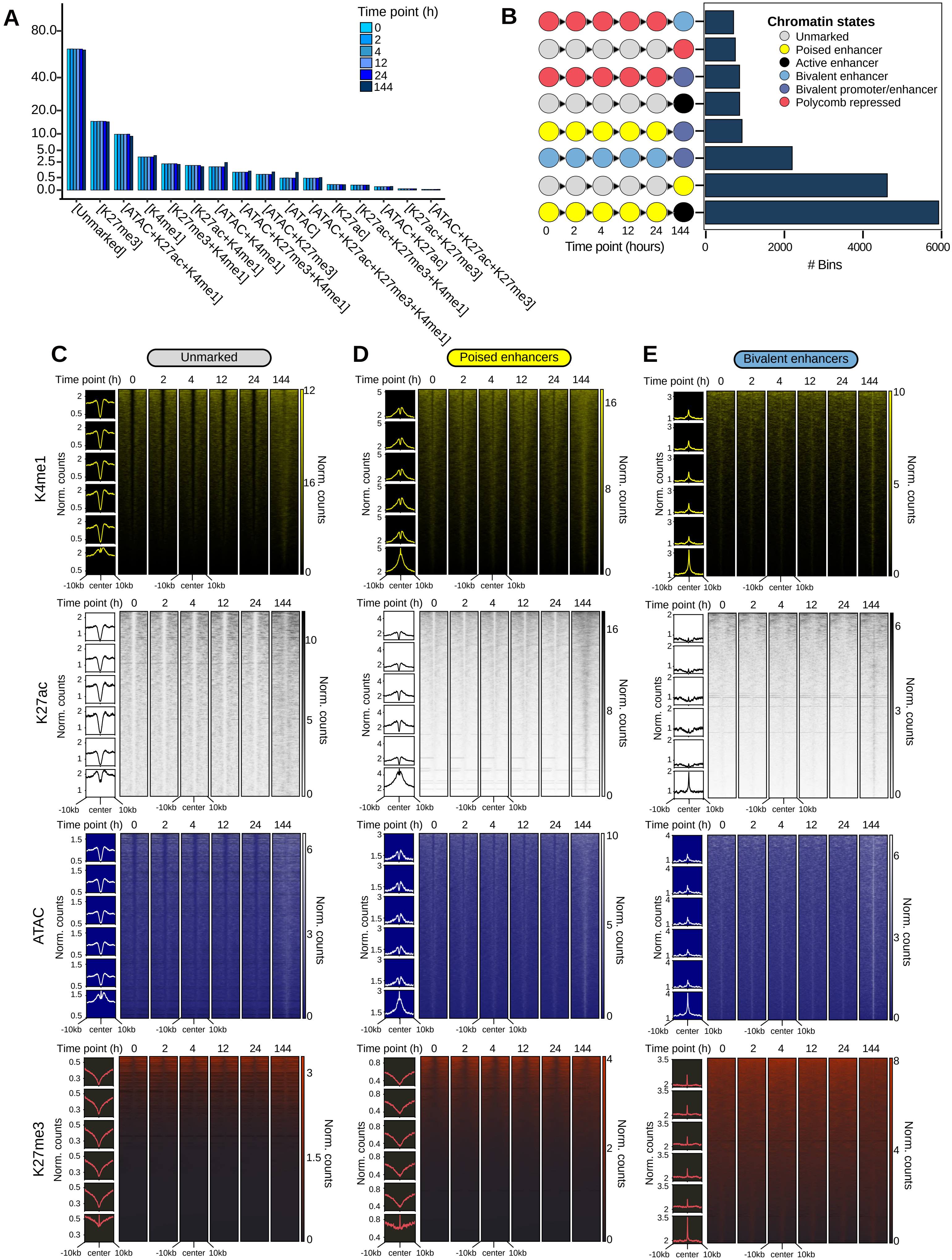

Supplementary Figure S4

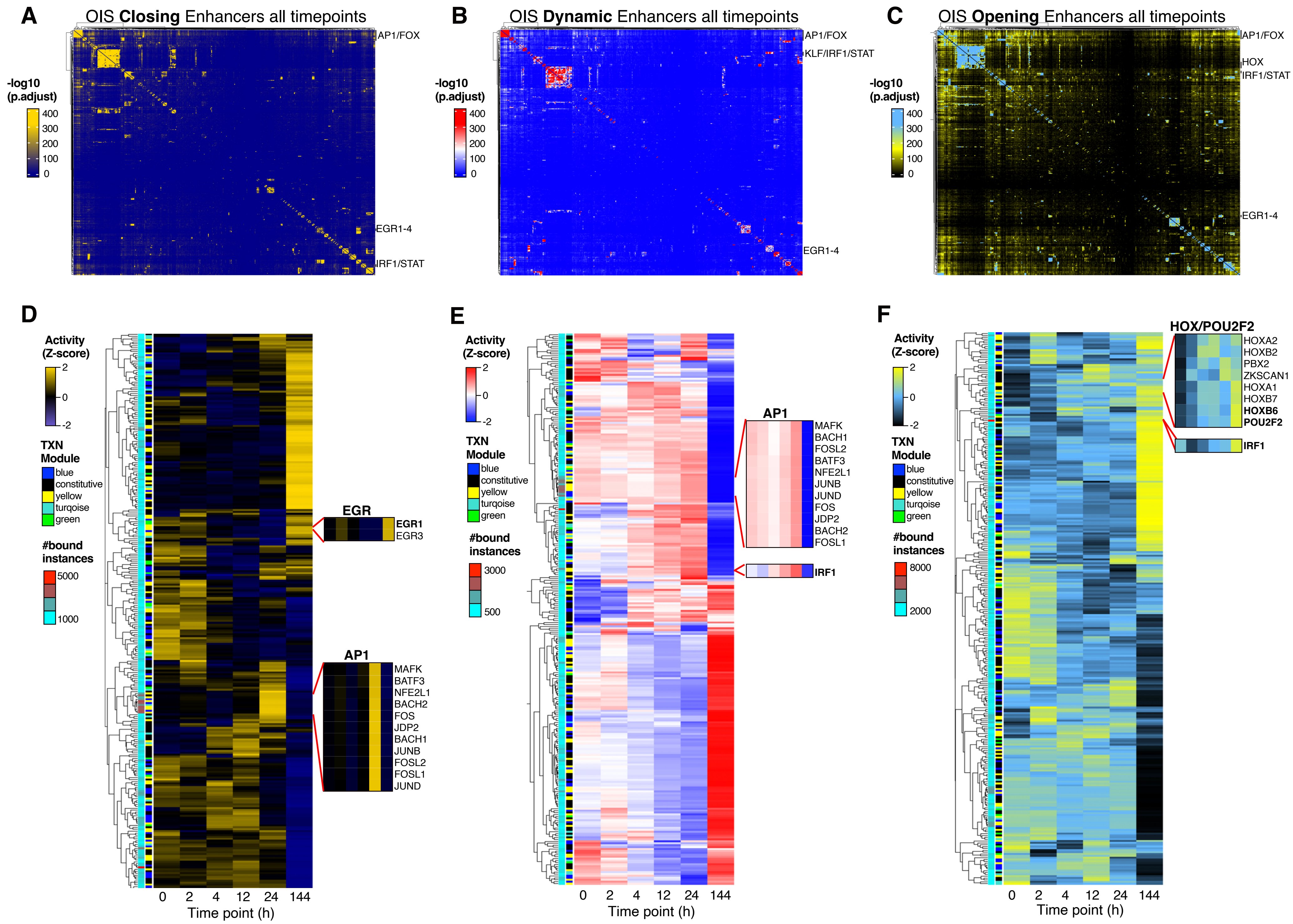

Supplementary Figure S5

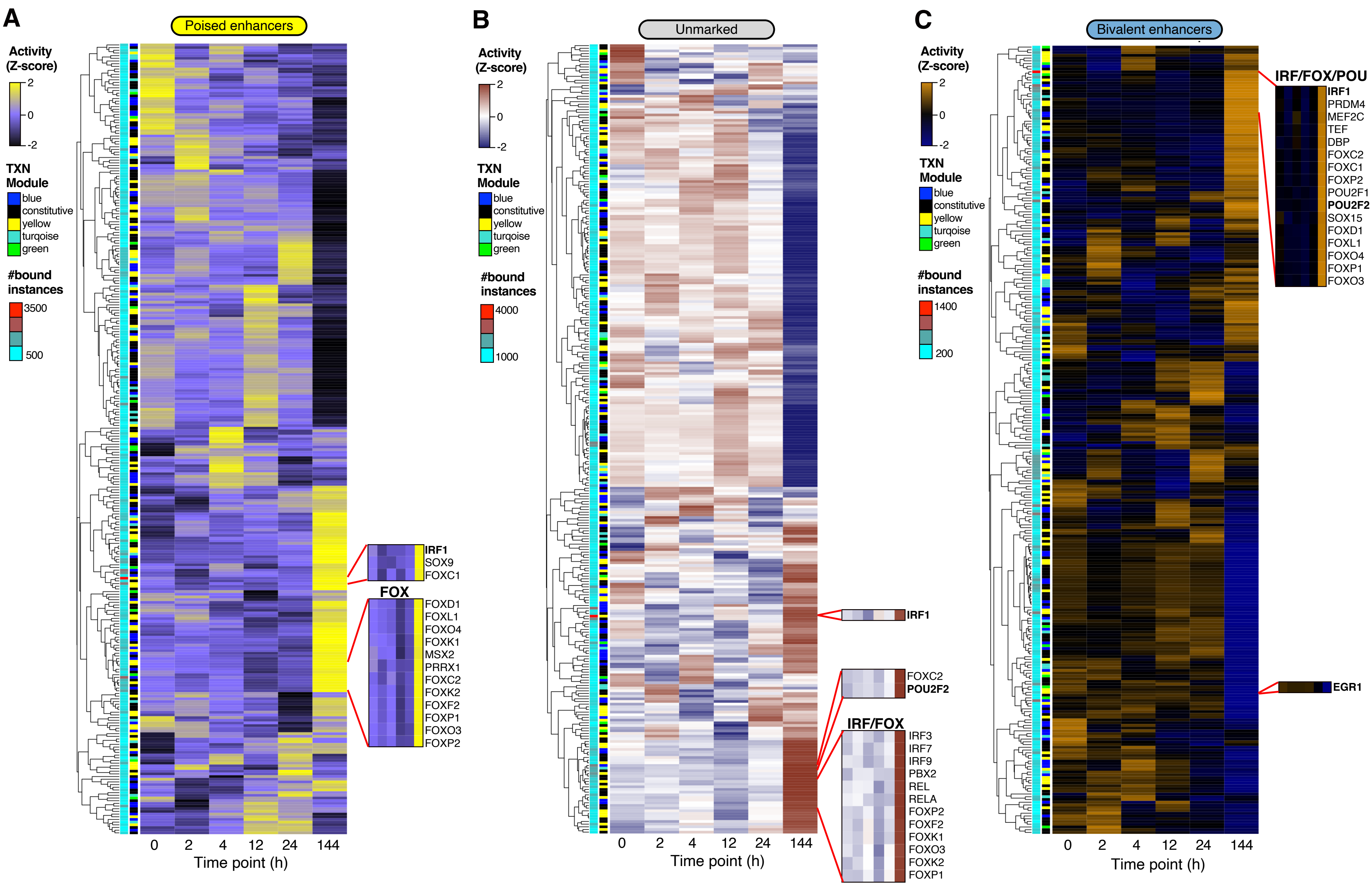

Supplementary Figure S6

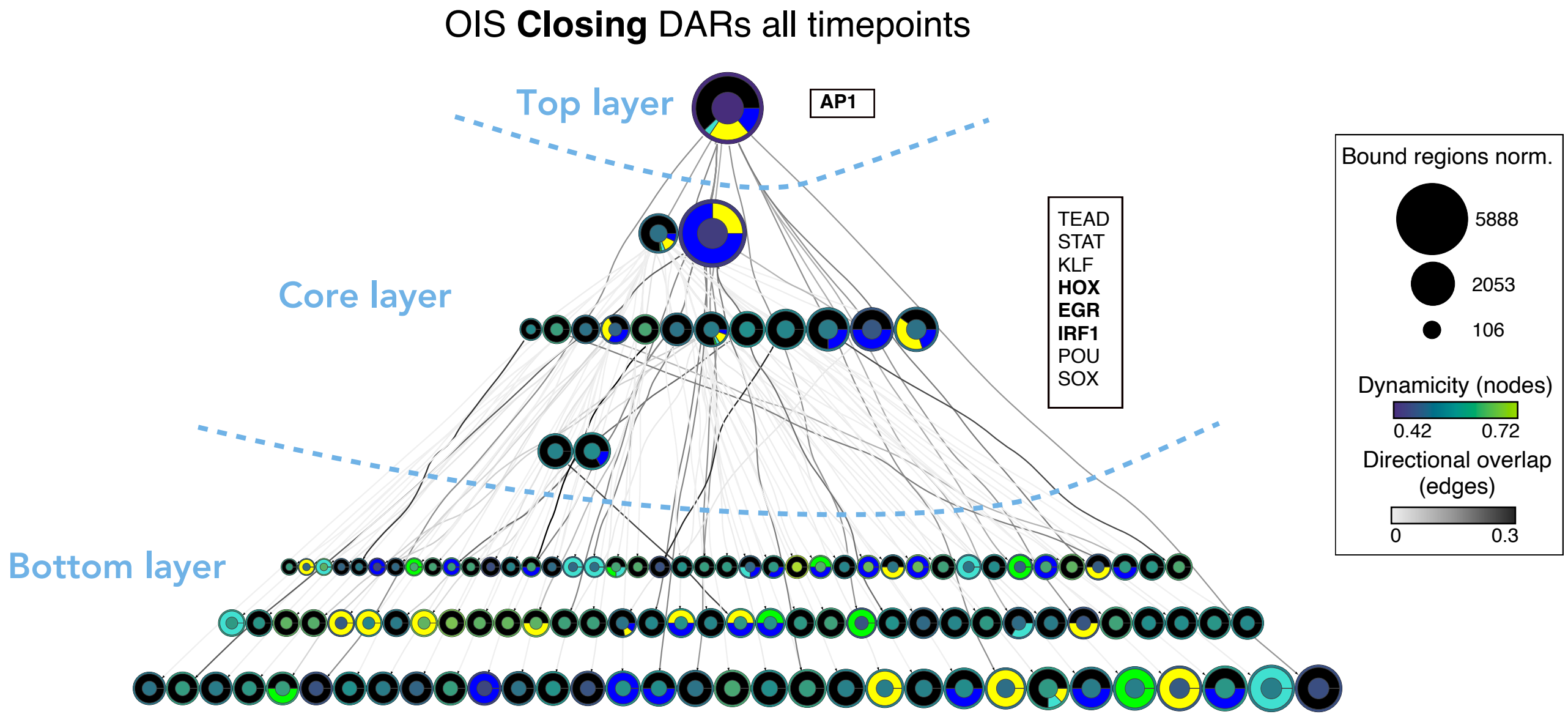

Supplementary Figure S7
